# Promoting behavior-related low back health in nurses by in-person and social media interventions in the workplace

**DOI:** 10.1186/s12912-022-01045-3

**Published:** 2022-10-05

**Authors:** Seyedeh-Somayeh Kazemi, Sedigheh-Sadat Tavafian, Claire E Hiller, Alireza Hidarnia, Ali Montazeri

**Affiliations:** 1 Department of Health Education and Health Promotion, Faculty of Medical Sciences, Tarbiat Modares University, Tehran, Iran; 2 School of Physiotherapy, Faculty of Health Sciences, University of Sydney, Sydney, Australia; 3 Faculty of Humanity Sciences, University of Sciences & Culture, ACECR, Tehran, Iran; 4 Health Metrics Research Center, Iranian Institute for Health Sciences Research, ACECR, Tehran, Iran

**Keywords:** PRECEDE-PROCEED model, In-person intervention, Social media intervention, Low back pain, Nurse

## Abstract

**Background:**

Nurses are put at high risk of work-related low back pain due to the nature of their work. The aim of this study was to develop and evaluate intervention based on the PRECEDE-PROCEED Model on promoting behaviors of low back health via two educational approaches.

**Methods:**

This study was a community randomized-controlled clinical trial. The educational content was developed with six modules: knowledge, attitude, self-efficacy, reinforcing factors, enabling factors, and behavior. Intervention was delivered by two modes: (1) in-person (n = 60) and (2) social media (n = 60). Data were evaluated by a self-designed questionnaire at baseline, 3, and 6 months. Baseline comparisons between groups were made with Mann-Whitney U Test and T-Test. Comparison of change scores between groups and two delivery types across the three time periods used the mixed between-within subject analysis of variance.

**Results:**

A total of 120 nurses received the allocated intervention. All educational component scores increased at 3-months in both groups. At the 6-month follow-up scores increased for enabling factors and behavior in the intervention group, while in the control group all scores increased except for attitude. Based on Bonferroni Post hoc analysis social media was more effective in knowledge, self-efficacy, reinforcing factors, and behavior than the in-person intervention.

**Conclusion:**

An educational program for low back health based on the PRECEDE-PROCEED model proved effective at improving all components. However, social media was more successful than in-person in the maintenance of behavior over the long term.

**Trial registration:**

IRCT20170313033054N2: 25-02-2018.https://www.irct.ir/trial/25598

## Background

The nurse is one of the key members of the health care team who has the appropriate scientific and practical capability for nursing care at different levels of prevention [1]. Due to the nature of their work nurses routinely execute activities that require lifting heavy loads, lifting patients, working in awkward postures, and transferring patients out of bed and from the floor [2, 3]. These work tasks put nurses at high risk of work-related musculoskeletal disorders (WMSDs) such as low back pain (LBP) with a lifetime prevalence ranging from 35 to 80% and associated with enormous socioeconomic and health costs to society [4–6]. There is wide scientific evidence that the prevalence of LBP is very high and it is the leading cause of sickness absence in healthcare workers, especially nurses working in hospitals [7], due to exposure to ergonomic and behavioral risk factors [7]. Therefore, promoting behaviors of low back health in nurses is essential.

Health-promoting behaviors are an international priority and a major challenge for healthcare providers in recent decades [8]. Although studies have shown ergonomic education to maintain an appropriate body posture at the workplace can reduce the prevalence of low back pain among nurses [9], but few studies have focused on changing risk behaviors such as not maintaining a correct posture, lack of predisposing, reinforcing and enabling factors to perform the behavior. Further, few have based their research on interventional models or theories, and few have focused on the course of low back pain after intervention or in other words, behavior maintenance for a long time [9–11].

The PRECEDE-PROCEED Model, which has been a cornerstone of health promotion practice for more than three decades, can help to guide the process of designing, implementing, and evaluating health behavior change programs [12]. This model can determine the causes of performing or not performing healthy behaviors and as well, determines the reinforcing and enabling factors in performing and maintaining healthy behavior [13]. Indeed, according to the PRECEDE-PROCEED model, three categories of factors change behavior: predisposing, reinforcing, and enabling factors [12]. Based on the existing literature [5, 14–16] and based on a qualitative study conducted by the researcher about the nurse’ experience of low back pain, factors affecting LBP health behaviors in the workplace [17] and also using appropriate education methods in the workplace, the educational intervention based on the model was designed and developed. The aim of this study was to develop and evaluate a theory-based educational intervention (PRECEDE-PROCEED Model) on promoting behaviors of low back health among nursing personnel with LBP. As well, we have compared two education approaches in-person and social media.

## Method

### Study design

As part of a trial [18], a community randomized-control trial study was conducted to develop and evaluate intervention based on the PRECEDE-PROCEED Model on promoting behaviors of low back health via two educational approaches including social media and in-person education approaches. The study was adopted from the declaration of Helsinki and received ethical approval from the Human Ethics Committee at the University of Tarbiat Modares, Tehran, Iran (IR. TUM. REC. 2017/545). Written informed consent was obtained from all participants.

### Setting

Hospitals affiliated to Mazandaran University of Medical Sciences in Sari, Iran.

### Participants

The study was conducted in two hospitals with a similar level of healthcare complexity. The participants were Iranian female nurses. Study design data were collected through interviews with participants. To recruit participants, an information session was held at each hospital, then participants were notified of the education time by posting announcements on the board and text messages. After obtaining informed consent, each participants completed the baseline questionnaires. Follow-up questionnaires were administered three and six months after the intervention. The questionnaires were anonymous. Participants were coded by the coordinator and they were identifiable by code of 1-300.

### Inclusion and exclusion criteria

Inclusion criteria for the study were: having work-related low back pain which was examined by a specialist in occupational medicine, having pain between 4 weeks to 3 months, having at least one year of nursing work experience, having access and skill to use a mobile phone, internet, and services online. Exclusion criteria were: pathological low back pain, having an illness (such as cancer, fractures, diabetes, cirrhosis, bipolar disorder, depression, schizophrenia etc.), and being pregnant.

### Intervention

#### Development of the educational program

**Fig. 1:**
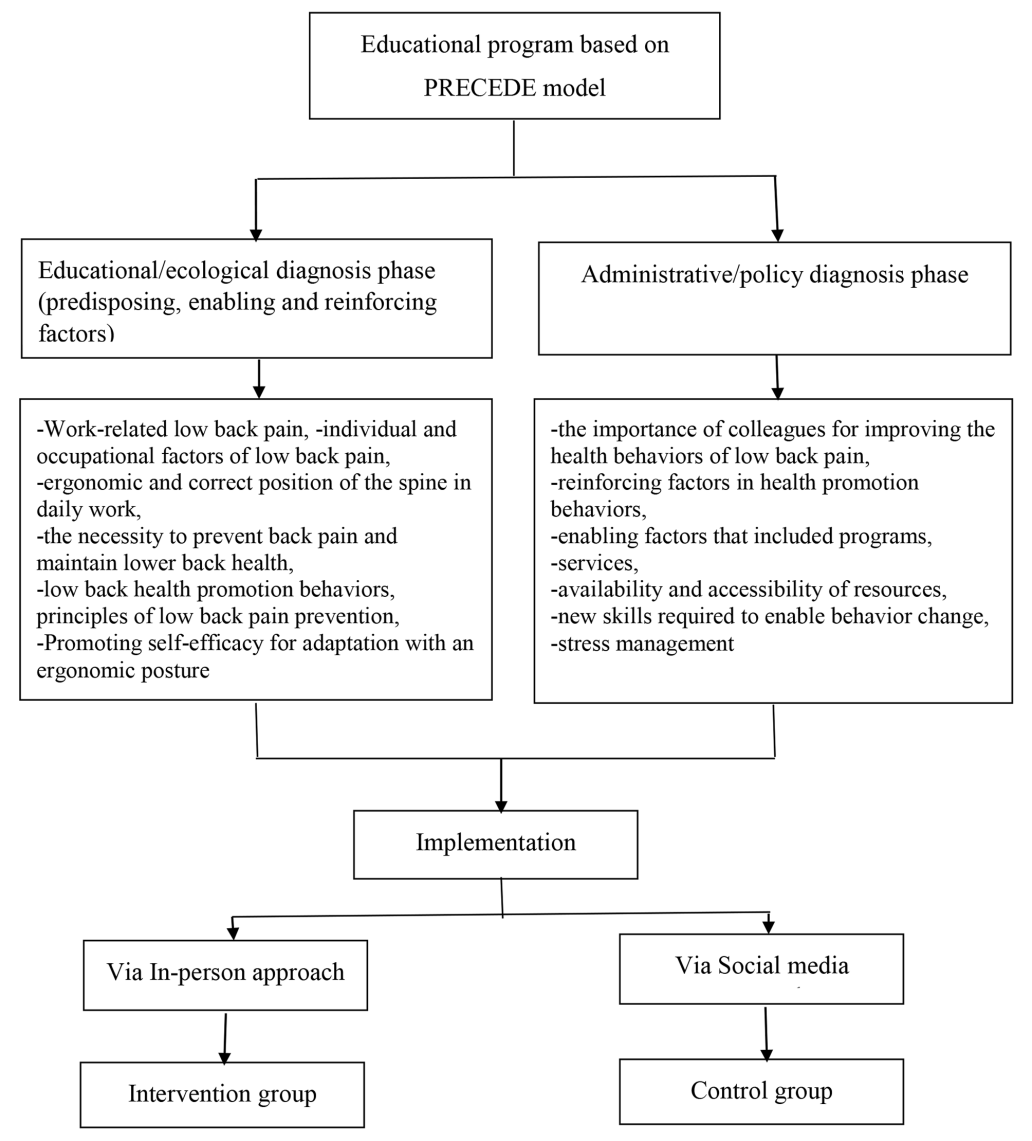
Flowchart of the development of the educational program

The main aim of the program was to promote behavior related to low back health in nurses who suffered from low back pain (Fig. 1). So, to found out what factors help to promote healthy behaviors and what factors prevented healthy behaviors in the workplace we carried out interviews with nursing personnel based on the educational/ecological diagnosis and administrative/policy diagnosis phases of the PRECEDE model [17]. Based on the educational/ecological diagnosis phase, we identified predisposing, enabling, and reinforcing factors [12] via an interview with nurses (n = 18), educational supervisors (n = 3), hospital manager (n = 1), and head nurse (n = 4). Indeed, they are factors that if modified, will most likely result in behavior change, as well as sustain it [12, 19]. Predisposing factors are those characteristics that motivate any recommended behavior before or during the happening of that behavior [12]. Predisposing factors include an individual’s knowledge, beliefs, values, attitudes, and self-efficacy. Enabling factors are those characteristics that facilitate action and include programs, services, availability and accessibility of resources, or new skills required to enable behavior change. Reinforcing factors are rewards or punishments following a consequence of any recommended behavior [12]. They could strengthen the motivation and some of the reinforcing factors include social support, peer support, or similar.

In the administrative/policy diagnosis phase [20], we investigated appropriate strategies for education implementation through interviews with the hospital managers, head nurses, nursing managers, and educational supervisors about hospital policies. This phase focused on the administrative and organizational concerns that must be addressed prior to program implementation such as assessment of available resources for nurses, development, and allocation of budgets to provide education, looking at organizational barriers, and coordination of the program with other departments [20].

Eventually, according to the results obtained from interviews and literature review [5, 14–16], the educational intervention was designed and developed. The educational content was developed with six modules: knowledge, attitude, self-efficacy, reinforcing, and enabling factors for promoting behavior of low back health.

The educational content included: definition of work-related low back pain, individual and occupational factors of low back pain, the ergonomic and correct position of the spine in daily work, the principles and necessity to prevent back pain and maintain lower back health, the procedures for improving low back pain, promoting self-efficacy for adaptation with an ergonomic posture. In addition, presentation of information about the importance of the role of colleagues as motivation, reinforcing factor in promoting healthy behaviors, enabling factors such as programs, services, availability and accessibility of resources, or new skills required to enable behavior change, and stress management. The details of the intervention are available in the implementation of the educational program section.

Before the education implementation, the website and educational content were evaluated by an educational technology expert, health education expert, educational management expert, and nurses. Educational material was authoritative scientific sources and used comprehensible language and a diversity of formats, including PowerPoint, photos, educational videos, and 3D animation.

### Validation of the educational program

The educational program was validated as part of a larger randomized controlled trial [18]. The educational program was provided to ten out-of-study nurses. They were asked to evaluate the program in terms of its comprehensibility, usability, and general features of the website and content. The CONSORT statement and the extension for randomized trials were used to describe the design of the study [21, 22].

### Implementation of educational program

The educational content was the same in both groups and just the delivery model was different. Participants in the intervention group (in-person) received the educational content in two 60 min sessions, and through lectures, role-playing, film, animation, questions and responses, and discussing nurse’s comments and experiences. Participants in the control group (social media) received educational content through interactive social media. For each participant in the control group, a proprietary username and password were created and instructions for social media log on and use were given and all logged on. The content of the education was uploaded to the site in two 60 min sessions and on 6-month duration. Nurses could download and save the educational content, film, and animation. Since the website was interactive, during this time, participants were able to share their comments, questions, and suggestions and receive feedback from the researcher. Also, participants were given a mobile number to contact the researcher if they had any problems, such as logging into the website or installing the app.

### Outcome

#### Primary outcome

The study’s main and primary outcome was promoting behavior related to low back health.

#### Secondary outcome

The secondary outcome was promoting knowledge, attitude, self-efficacy, reinforcing factors, enabling factors related to behavior.

### Instrument

A self-design questionnaire (Occupational Back Pain Prevention Behavior Questionnaire) was an instrument for measuring the constructs of the PRECEDE-PROCEED Model such as educational/ecological diagnosis (predisposing, reinforcing, and enabling factors) and administrative/policy diagnosis (educational strategies). The instrument questions were designed based on a semi-structured interview of nurses. This questionnaire consisted of 30 items and six components of health status including knowledge (4-item), attitude (5-item), self-efficacy (6-item), reinforcing factor (5-item), enabling factor (7-item), and behavior (3-item). To calculate each subscale or total score for the Occupational Back Pain Prevention Behavior Questionnaire first we added raw scores and linearly transferred them to a score from 0 to 100. Items were scored with the Likert spectrum. The Likert scale is a five-point scale from 1 to 5. The greater the score showed the better condition in nurses. The validity and reliability of this questionnaire were confirmed by Cronbach’s alpha of 0.92 [23]. The questionnaire was completed at baseline, 3-month, and 6-month follow-up. The questionnaire took 15 to 20 min to complete.

### Sample size

The sample size was calculated based on the Pokak formula and at 95% confidence level and 80% power and with a mean difference and standard deviation of 5.44 (2.55) before and after the intervention, 55 were considered in each hospital [4] and with 10% chance of dropout, 60 nurses were estimated to be required in each hospital.

### Randomization

Hospitals were allocated to intervention group 1 and the intervention group 2 by draw. Then nursing staff with low back pain at each hospital were selected by simple randomization. Each hospital sent a list of nurses’ IDs to the study coordinator. The coordinator coded the IDs to numbers 1-300. A random number table using these numbers was generated. The coordinator contacted the nurses in order of the random table, and then assessed them for eligibility and consent. This process continued until the sample size of 60 was reached.

This study was a single-blind trial and the participants did unaware of the intervention they receive. To ensure allocation concealment, randomization to groups was undertaken by a blinded remote investigator not involved in recruitment. Participants selected from two separate hospitals. The researcher has explained the aim of the study in the intervention group and in the control group by a briefing meeting. Participants had the possibility to talk with each other about the treatment in the control group by the website.

### Statistical analysis

The Shapiro-Wilk test was used to determine the normality of the data. To compare baseline scores of knowledge, attitude, self-efficacy, reinforcing factors, enabling factors and behavior, Mann-Whitney U Test was used for non-normal data and a T-Test for normal data. We planned to use a mixed-between-within subject analysis for each factor, but as they were not all normally distributed, so change scores were calculated. To compare change scores of the six components between groups over time and also to compare the two types of delivery, a mixed between-within subject ANOVA of variance was conducted with post-hoc Bonferroni analysis on significant results. Additionally, Pearson-correlation was used to determine the relationship between all factors and behavior. All analysis was with SPSS IBM Statistics version 23.

## Results

**Fig. 2:**
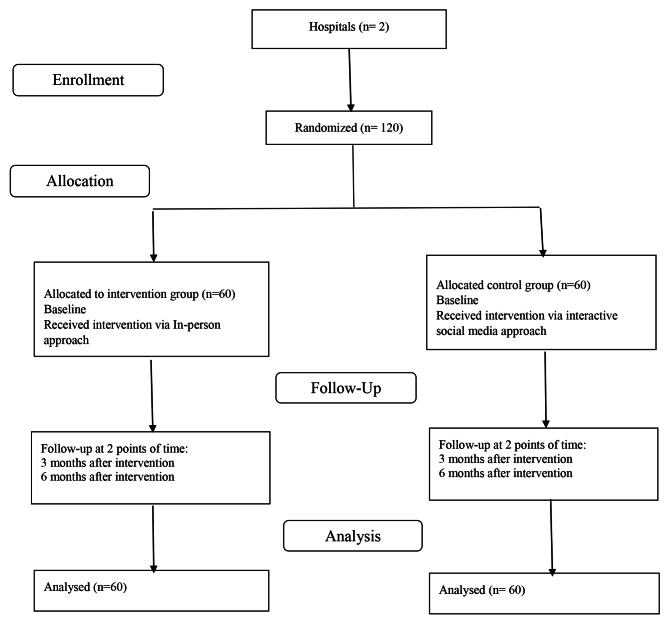
Flowchart and overview of the trial

An educational intervention was developed based on six components: knowledge, attitude, self-efficacy, reinforcing factors, enabling factors and behavior. It was modified for an in-person and social media delivery. Validation was undertaken with 120 female nurses (mean age 36.50 ± 5.79) years, mean height 161.93 ± 6.68 cm, mean weight 66.08 ± 11.65 kg and mean BMI 25.08 ± 3.21. Figure 2 displays the flowchart and overview of the trial study. There were no significant differences between the two groups at baseline in any of the six components (Table 1).

**Table 1:** Median scores of predictive factors in two groups at baseline

	In-person group		Social media group		
	**Median**	**IQ range**	**Median**	**IQ range**	p*
Knowledge*	50	50–75	50	50–75	0.98
Attitude*	55	50-63.75	55	50–60	0.54
Self-efficacy*	50	41.67–62.50	52.08	45.83–62.50	0.98
Reinforcing factor	55	40–65	50	40–65	0.90
Enabling factor	42.86	32.14–57.14	44.64	32.14–57.14	0.89
Behaviour*	50	33.33–58.33	50	33.33–58.33	0.92

* Mann-Whitney U Test. All others by T-Test

Table 2 displays the six components of the educational intervention over the 3 and 6-month follow-up in both groups.

**Table 2:** The scores of predictive factors at 3- points of time in two groups

	In-person group			Social media group		
	Baseline	3-month	6-month	Baseline	3-month	6-month
	M ± SD	M ± SD	M ± SD	M ± SD	M ± SD	M ± SD
Knowledge	56.25 ± 22.37	90 ± 13.96	80.83 ± 15.51	56.67 ± 23.85	93.33 ± 12.06	93.75 ± 1091
Attitude	54.75 ± 8.1	84.92 ± 11.4	84.50 ± 11.33	55.08 ± 7.45	86.50 ± 10.3	86.33 ± 9.64
Self-efficacy	52.71 ± 13.65	60.56 ± 13.23	59.86 ± 12.92	52.22 ± 13.56	65.90 ± 13.32	67.36 ± 15.85
Reinforcing factor	53.67 ± 16.46	60.67 ± 14.30	60.25 ± 15.33	53.33 ± 15.34	62.25 ± 16.96	66 ± 16.84
Enabling factor	45.24 ± 17.44	47.20 ± 15.33	50 ± 11.65	44.82 ± 16.51	51.85 ± 14.18	53.45 ± 9.64
Behaviour	49.58 ± 19.31	57.78 ± 14.38	59.31 ± 8.55	49.31 ± 19.12	62.92 ± 12.69	71.53 ± 6.38

Based on calculated change scores, all components increased over the 3-months after intervention in both groups. But over the 6-month follow-up increased scores for enabling factors and behavior occurred in the intervention group (in-person intervention), while in the control group (social media intervention) all components except for attitude improved (Table 3). Based on Bonferroni Post hoc analysis there was a difference between the delivery types; social media approach was more effective and successful in knowledge (p = .01), self-efficacy (p = .03), reinforcing factor (p = .05) and behavior (p = .001) than the in-person approach. There just was a positive moderate correlation between self-efficacy and behavior, r = .540, p ≤ .0001. Thus with increasing self-efficacy, behaviors related to low back health increased.

**Table 3:** Comparison of change scores of predictive factors in two groups at 3- points of time

	(Baseline and 3-month follow-up)		(3-month and 6-month follow-up)		P*	Eta
	In-person	Social media	In-person	Social media		
	M ± SD	M ± SD	M ± SD	M ± SD		
Knowledge	33.75 ± 24.27	36.66 ± 25.40	-9.17 ± 19.51	0.42 ± 16.90	0.01	0.050
Attitude	30.16 ± 14.02	31.41 ± 12.38	− 0.41 ± 15.19	− 0.16 ± 16.07	0.52	0.003
Self-efficacy	7.84 ± 20.96	13.68 ± 20.61	− 0.69 ± 18.12	1.46 ± 21.57	0.03	0.039
Reinforcing factor	7.00 ± 21.43	8.91 ± 21.59	− 0.41 ± 21.84	6.33 ± 21.64	0.05	0.032
Enabling factor	1.96 ± 25.91	7.02 ± 23.32	2.79 ± 17.84	1.61 ± 16.78	0.30	0.009
Behaviour	8.20 ± 25.04	13.61 ± 23.26	1.53 ± 14.59	8.61 ± 13.37	0.001	0.095

* Based on mixed between-within subject analysis of variance

## Discussion

An educational program based on the PRECEDE-PROCEED model for promoting behavior related to low back health was successfully developed and implemented via two methods, in-person and social media. Evaluation of the program showed that all components improved over the 3-month follow-up for both delivery types and over the 6-month follow-up for the social media intervention, while two components improved over the 6-month follow-up for the in-person intervention (enabling factors and behavior).

Our educational program was in line with previous literature, which acknowledged that the use of community-based participatory research models to guide intervention development can contribute to more engaging and effective health behavior interventions [24–26]. A fundamental assumption of the PRECEDE-PROCEED model is the active participation of its intended audience that is that the participants will take an active part in defining their own problems, establishing their goals and developing their solutions [12]. This is supported by a systematic review which advocated for the use of social or behavioral theories in the prevention of musculoskeletal injuries [27]. The results of Ebadifard’ et al’s., study indicated the effectiveness of an in-person educational intervention based on a PRECEDE-PROCEED model combined with self-management theory improved self-care behaviors in patients [28].

In our study, nurses’ knowledge and attitude towards LBP increased over the 3-month period for both interventions. Nurses are knowledgeable regarding health-promoting activities such as physical activity, stress management, and maintaining healthy relationships. However, this knowledge may not translate into nurses’ own self-care or health behavior [29]. Our result was supported by Janssens’ study which showed an increase in knowledge and attitude of the care staff following a healthcare program [30]. As well as, other studies showed improvement of knowledge and attitude (HPV Vaccination, Anemia, self-care, pain management, physical activity, oral health) following educational intervention delivered by in-person and web-based [30–35]. Similarly the result of McNamara’ study et al., showed the acute pain educational program intervention improved nurses’ knowledge and attitudes towards pain assessment and management over the 6 weeks [36]. At the 6-month follow-up, nurses’ knowledge and attitude in the in-person and attitude in the social media group was reduced. Schaller et al. compared two educational methods in physical activity (movement coaching; phone and web and low-intensity control; using two oral presentations). They found that at 6-month follow-up there were no statistically significant between-group differences in physical activity [37]. It seems face-to-face education is not sufficient for the enhancement of knowledge. It is essential to use a complementary educational method can be used at any time and place and have the ability to remind and repeat. It seems social media can be more effective in the long-term due to the mentioned capabilities.

The educational program led to increasing self-efficacy over 3-months. Maintenance at 6-months was more effective with the program being delivered via social media. In a study by George et al, the findings suggested the use of two types of in-person education (Dedicated Education Unit and Traditional Clinical Education) had a significant increase in self-efficacy scores post clinical education in both groups [38]. Also Thompson’ study showed that online intervention in nursing students were associated with a statistically significant increase in self-efficacy on bullying behavior [39]. At 6-months follow-up, self-efficacy decreased among the in-person group. It seems that using a program with easy access will help to maintain self-efficacy. However, in-person group did not have access to educational materials after the intervention ended.

In our study, there was a moderate positive correlation between self-efficacy and behavior. Higher self-efficacy score indicated increased health behavior. These results were supported by Fida’ study et al., who found self-efficacy was an important protective factor against negative behavior in the workplace [40]. Indeed, self-efficacy is defined as one’s perceived capability for learning or performing actions at designed levels [41]. Self-efficacy is hypothesized to influence behaviors and environments and in turn be affected by them [41, 42]. People with higher levels of self-efficacy tend to choose more challenging tasks, persist in personal behaviors when encountering difficulties, confront adversities with courage, and have higher levels of confidence [43]. Self-efficacy is extensively applied in health behavior-related fields, to patients suffering from chronic pain, workplace incivility, and burnout in nursing [40]. A systematic review demonstrates that interventions that modify attitudes, norms, and self-efficacy are effective in promoting health behavior change [44].

A meta-analysis study showed that for pain intensity, evidence indicated there was a clinically important effect of e-Health-based self-management programs for relieving pain both at immediate and short-term follow-ups and disability at immediate follow-up [45]. Zachary’ study discovered the effect of an E-learning module in addition to attitudes, confidence and knowledge, on clinical skills chronic low back pain in older adults [46]. Nursing leaders can be concerned with improving participation in health-promoting behaviors not only because it is a workplace health issue, but because it is potentially a financial and patient safety issue. Fortunately, we saw an increase in the enabling factors over the 3 and 6 months’ follow-up in both groups. This suggests that management factors and policies can play a major role in the adoption and promotion of health behaviors. Managers can provide the environment for exercise facilities and comfortable spaces for managing workplace stress. Ross’ study supports the results of our study. The results provided strategies in the nursing workplace to improve the health of staff nurses by increasing health-promoting behaviors [29]. The social relationship between colleagues, reward, and satisfaction from the outcome of adopting the behavior, and the role of colleagues are effective factors in improving health behaviors. The effect of these factors has observed over the 3-months follow-up in both groups. But over the 6-months follow-up decreased in the in-person group. We provided the certification to participate in the intervention as a reward. After the intervention, we encouraged participants to maintain the behavior through the website and recalled the role of colleagues in promoting the behavior. Perhaps because of the lack of interaction at the end of in-person education, the role of the reinforcing factors was diminished and individuals had not acted as incentives for one another.

The result showed behavior score was improved over 3-month after the intervention. This is in line with the findings of Maghbouli et al., from the effect of an educational intervention in healthy behaviors of the nursing students to prevent LBP [47]. Even a qualitative study also noted interactive websites for people with chronic pain lead to improved health literacy, self-efficacy, empowerment, improvements in physical exercise and overall quality of life [48]. However, in our study, social media intervention was more successful in improving behavior than in-person intervention over the 6-month follow-up. Literature showed the mobile-web program (FitBack) in adults performed better on behavior of self-management of low back pain, and worksite outcomes at 4-month follow-up. Further, indicated greater improvement at 4-month follow-up on patient activation, behavior, and attitudes toward pain [36].

## Limitations

One of the limitations of this study was the differences between educational exposures in two groups. In the in-person group, we were not sure that nurses performed health behaviors in the workplace. But in the social media group, we were aware through nurses’ feedback on the website. The study participants were female nurses. Educational intervention for male nurses is also recommended in the next studies.

## Conclusion

Nurses’ knowledge of the importance of health behaviors regarding LBP does not mean they apply healthy behaviors to themselves. The model-based educational intervention for LBP proved effective at improving knowledge, attitude, self-efficacy, reinforcing factors, enabling factors, and behavior immediately and after 3 months. However, social media was more successful in the maintenance of behavior over the long-time. Changing behaviors related to low back health need suitable context and easy access to education through the best channels, which appear interactive social media to be appropriate.

## Data Availability

The datasets used and/or analysed during the current study are available from the corresponding author upon reasonable request.

## Abbreviations

**WMSDs**: Work-Related Musculoskeletal Disorders
**LBP**: Low Back Pain

## References

[1] Halcomb E, Williams A, Ashley C, McInnes S, Stephen C, Calma K, James S. The support needs of Australian primary health care nurses during the COVID-19 pandemic. Journal of nursing management. 2020 Oct;28(7):1553–60. 10.1111/jonm.13108. 10.1111/jonm.13108 32713047

[2] Járomi M, Kukla A, Szilágyi B, Simon-Ugron Á, Bobály VK, Makai A (2018). Back School programme for nurses has reduced low back pain levels: A randomised controlled trial. J Clin Nurs.

[3] Asadi P, Kv M, Zsm Z, Zohrevandi B. The prevalence of low back pain among nurses working in Poursina hospital in Rasht, Iran. Asadi P, Monsef Kasmaei V, Zia Ziabari SM, Zohrevandi B The prevalence of low back pain among nurses working in Poursina hospital in Rasht, Iran Journal of Emergency Practice and Trauma 2016; 2(1):11 – 5. 2016;2(1):11 – 5.

[4] Pakbaz M, Hosseini MA, Aemmi SZ, Gholami S (2019). Effectiveness of the back school program on the low back pain and functional disability of Iranian nurse. J Exerc rehabilitation.

[5] Ovayolu O, Ovayolu N, Genc M, Col-Araz N (2014). Frequency and severity of low back pain in nurses working in intensive care units and influential factors. Pakistan J Med Sci.

[6] Moreira RF, Sato TO, Foltran FA, Silva LC, Coury HJ (2014). Prevalence of musculoskeletal symptoms in hospital nurse technicians and licensed practical nurses: associations with demographic factors. Braz J Phys Ther.

[7] Serra C, Soler-Font M, García AM, Peña P, Vargas-Prada S, Ramada JM (2019). Prevention and management of musculoskeletal pain in nursing staff by a multifaceted intervention in the workplace: design of a cluster randomized controlled trial with effectiveness, process and economic evaluation (INTEVAL_Spain). BMC Public Health.

[8] Myers RE (2010). Promoting healthy behaviors: how do we get the message across?. Int J Nurs Stud.

[9] Jradi H, Alanazi H, Mohammad Y (2020). Psychosocial and occupational factors associated with low back pain among nurses in Saudi Arabia. J Occup Health.

[10] Keogh A, Tully MA, Matthews J, Hurley DA (2015). A review of behaviour change theories and techniques used in group based self-management programmes for chronic low back pain and arthritis. Man Therap.

[11] Richardson A, McNoe B, Derrett S, Harcombe H (2018). Interventions to prevent and reduce the impact of musculoskeletal injuries among nurses: A systematic review. Int J Nurs Stud.

[12] Green L, Kreuter M (2005). Health program planning: an educational and ecological approach.

[13] Glanz K, Rimer BK, Viswanath K. Health behavior: Theory, research, and practice. John Wiley & Sons; 2015. p. 5.

[14] Tosunoz IK, Oztunc G (2017). Low back pain in nurses. Int J Caring Sci.

[15] Asuquo EG, Tighe SM, Bradshaw C (2021). Interventions to reduce work-related musculoskeletal disorders among healthcare staff in nursing homes; An integrative literature review. Int J Nurs Stud Adv.

[16] El-Soud AMA, El-Najjar AR, El-Fattah NA, Hassan AA (2014). Prevalence of low back pain in working nurses in Zagazig University Hospitals: an epidemiological study. Egypt Rheumatol Rehabilitation.

[17] Kazemi S-S, Tavafian S-S, Hidarnia A, Montazeri A. Consequences and factors affecting work-related low back pain among nursing professionals: A qualitative study. Payesh (Health Monitor). 2019;18(3):291–303. URL: http://payeshjournal.ir/article-1-1085-en.html.

[18] Kazemi SS, Tavafian S-S, Montazeri A (2019). The social media intervention for lower back pain education study (SMILE): a protocol for a randomized trial to reduce occupational low back pain in nursing professionals. Int J Musculoskelet Pain Prev.

[19] Zhu X, Song C, Lu T, Jin M. Proposal and Efficacy of a Nurse-Led Pain Management Model for Neurointensive Care Based on the Precede-Proceed Model. Computational and Mathematical Methods in Medicine. 2022;2022. 10.1155/2022/5686433. 10.1155/2022/5686433 PMC9377952 35979046

[20] Green L, Ottoson J, Novick L, Morrow C, Mays G (2008). Public Health Education and Health Promotion.

[21] Schulz KF, Altman DG, Moher D, Group C (2010). CONSORT 2010 statement: updated guidelines for reporting parallel group randomized trials. Ann Intern Med.

[22] Altman DG, Schulz KF, Moher D, Egger M, Davidoff F, Elbourne D (2001). The revised CONSORT statement for reporting randomized trials: explanation and elaboration. Ann Intern Med.

[23] Kazemi SS, Tavafian SS, Hidarnia A, Montazeri A (2020). Development and validation of an instrument of occupational low back pain prevention behaviours of nurse. J Adv Nurs.

[24] Ashton LM, Morgan PJ, Hutchesson MJ, Rollo ME, Collins CE (2017). Feasibility and preliminary efficacy of the ‘HEYMAN’healthy lifestyle program for young men: a pilot randomised controlled trial. Nutr J.

[25] Whatnall M, Patterson A, Hutchesson M (2019). A brief web-based nutrition intervention for young adult university students: Development and evaluation protocol using the precede-proceed model. JMIR Res protocols.

[26] Calano BJD, Cacal MJB, Cal CB, Calletor KP, Guce FICC, Bongar MVV (2019). Effectiveness of a community-based health programme on the blood pressure control, adherence and knowledge of adults with hypertension: A PRECEDE‐PROCEED model approach. J Clin Nurs.

[27] Gabriel EH, McCann RS, Hoch MC (2019). Use of social or behavioral theories in exercise-related injury prevention program research: a systematic review. Sports Med.

[28] Azar FE, Solhi M, Darabi F, Rohban A, Abolfathi M, Nejhaddadgar N (2018). Effect of educational intervention based on PRECEDE-PROCEED model combined with self-management theory on self-care behaviors in type 2 diabetic patients. Diabetes & Metabolic Syndrome: Clinical Research & Reviews.

[29] Ross A, Bevans M, Brooks AT, Gibbons S, Wallen GR (2017). Nurses and health-promoting behaviors: Knowledge may not translate into self-care. AORN J.

[30] Janssens B, Vanobbergen J, Lambert M, Schols J, De Visschere L (2018). Effect of an oral healthcare programme on care staff knowledge and attitude regarding oral health: a non-randomised intervention trial. Clin Oral Invest.

[31] Dixon BE, Zimet GD, Xiao S, Tu W, Lindsay B, Church A, et al. An educational intervention to improve HPV vaccination: a cluster randomized trial. Pediatrics. 2019;143(1). 10.1542/peds.2018-1457 30530637

[32] Ernawati E, Baso YS, Hidayanty H, Syarif S, Aminuddin A, Bahar B (2022). The effects of anemia education using web-based she smart to improve knowledge, attitudes, and practice in adolescent girls. Int J Health Med Sci.

[33] Peyman N, Rezai-Rad M, Tehrani H, Gholian-Aval M, Vahedian-Shahroodi M, Heidarian Miri H (2018). Digital Media-based Health Intervention on the promotion of Women’s physical activity: a quasi-experimental study. BMC Public Health.

[34] Latina R, Mauro L, Mitello L, D’Angelo D, Caputo L, De Marinis MG (2015). Attitude and knowledge of pain management among Italian nurses in hospital settings. Pain Manage Nurs.

[35] Sayakhot P, Carolan-Olah M, Steele C (2016). Use of a web-based educational intervention to improve knowledge of healthy diet and lifestyle in women with gestational diabetes mellitus compared to standard clinic-based education. BMC Pregnancy Childbirth.

[36] McNamara MC, Harmon DC, Saunders J (2012). Effect of education on knowledge, skills and attitudes around pain. Br J Nurs.

[37] Schaller A, Petrowski K, Pfoertner T-K, Froboese I (2017). Effectiveness of a theory-based multicomponent intervention (Movement Coaching) on the promotion of total and domain-specific physical activity: a randomised controlled trial in low back pain patients. BMC Musculoskelet Disord.

[38] George LE, Locasto LW, Pyo KA, Cline TW (2017). Effect of the dedicated education unit on nursing student self-efficacy: A quasi-experimental research study. Nurse Educ Pract.

[39] Thompson R, George LE (2016). Preparing new nurses to address bullying: The effect of an online educational module on learner self-efficacy. Medsurg Nurs.

[40] Fida R, Laschinger HKS, Leiter MP (2018). The protective role of self-efficacy against workplace incivility and burnout in nursing: A time-lagged study. Health Care Manage Rev.

[41] Bandura A (1997). The nature and structure of self-efficacy. Self-efficacy: the exercise of control.

[42] Bandura A (1986). The explanatory and predictive scope of self-efficacy theory. J Soc Clin Psychol.

[43] Banciura A (1977). Self-efficacy: Toward a unifying theory of behavior change. Psychol Rev.

[44] Sheeran P, Maki A, Montanaro E, Avishai-Yitshak A, Bryan A, Klein WM (2016). The impact of changing attitudes, norms, and self-efficacy on health-related intentions and behavior: A meta-analysis. Health Psychol.

[45] Du S, Liu W, Cai S, Hu Y, Dong J (2020). The efficacy of e-health in the self-management of chronic low back pain: a meta analysis. Int J Nurs Stud.

[46] Jacobs ZG, Elnicki DM, Perera S, Weiner DK (2018). An E-learning Module on Chronic Low Back Pain in Older Adults: Effect on Medical Resident Attitudes, Confidence, Knowledge, and Clinical Skills. Pain Med.

[47] Maghbouli R, Kazemi S-S, Jamshidi AR. The effect of an educational Intervention on low back pain preventive behavior among nursing students: A pre-posted designed study. International Journal of Musculoskeletal Pain Prevention. 2018;3(4):102-6. URL: http://ijmpp.modares.ac.ir/article-32-32305-en.html.

[48] Merolli M, Gray K, Martin-Sanchez F, Schulz P, editors. Expert insights on the design and implementation of interactive patient websites for people with chronic pain. HIC; 2014. doi:10.3233/978-1-61499-427-5-110. 25087536

